# Mutation screening of the *USH2A* gene reveals two novel pathogenic variants in Chinese patients causing simplex usher syndrome 2

**DOI:** 10.1186/s12886-020-01342-y

**Published:** 2020-02-24

**Authors:** Chenhao He, Xinyu Liu, Zilin Zhong, Jianjun Chen

**Affiliations:** 1 Birth defect group, Translation Research Institute of Brain and Brain-Like Intelligence, Shanghai Fourth People’s Hospital Affiliated to Tongji University School of Medicine, Shanghai, 200081 China; 2 Department of Medical Genetics, Tongji University School of Medicine, Shanghai, 200092 China; 3 Department of Pediatrics of Shanghai Tenth People’s Hospital, Tongji University School of Medicine, Shanghai, 200092 China; 4 Department of Ophthalmology of Shanghai Tenth People’s Hospital, and Tongji Eye Institute, Tongji University School of Medicine, Shanghai, 200092 China; 5 Birth defect group, Medical wing building, Tongji University School of Medicine, 1239 SipingRoad Yangpu District, Shanghai, 200092 China

**Keywords:** Usher syndrome, *USH2A*, Mutations, Sequence variants

## Abstract

**Background:**

Usher syndrome (USH) is the most prevalent cause of the human genetic deafness and blindness. USH type II (USH2) is the most common form of USH, and *USH2A* is the major pathogenic gene for USH2*.* For expanding the spectrum of USH2A mutations and further revealing the role of USH2A in USH2, we performed the USH2A gene variant screening in Chinese patients with USH2.

**Methods:**

Genomic DNA was extracted from peripheral blood of unrelated Chinese USH2 patients, we designed specific primers for amplifying the coding region (exons 2–72) of the *USH2A* gene. Sanger sequencing was used to study alleles. Silico prediction tools were used to predict the pathogenicity of the variants identified in these patients.

**Results:**

Five heterozygous pathogenic variants were detected in four patients. Two patients were found to have two-mutations and two patients only have one. Two novel variants c.4217C > A (p.Ser1406X) and c.11780A > G (p.Asp3927Gly)) were predicted deleterious by computer prediction algorithms. In addition, three reported mutations (c.8559-2A > G, c.8232G > C and c.11389 + 3A > T) were also found in this study.

**Conclusions:**

We identified five heterozygous pathogenic variants in the *USH2A* gene in Chinese patients diagnosed with Usher syndrome type 2, two of which were not reported. It expands the spectrum of *USH2A* variants in USH.

## Background

Usher syndrome (USH), an autosomal recessive disorder, is a clinically and genetically heterogeneous disease. USH is characterized by retinitis pigmentosa (RP), bilateral sensorineural hearing impairment and intact vestibular responses [1]. It is the most prevailing cause of the human hereditary deafness and blindness. In worldwide, the general prevalence of USH approximately ranges from 3.3 to 6.4 per 100,000 individuals [2]. Up to now, it is unavailability of a therapy for the USH.

Clinically, according to the severity and progression of vision and hearing loss of patients, USH classified into USH type I (USH1), USH type II (USH2), and USH type III (USH3) [3]. Besides, approximately 20–30% cases are categorized as atypical USH. USH1 is the most serious form in the three types, patients with USH1 have congenital profound hearing loss and begin to lose their vision early in life. Different from the USH1 patients defined as having congenital deafness and blindness within the first decade of life, patients with USH2 exhibit congenital mild-moderate hearing and vision loss in the second decade of life, and generally show normal vestibular function in all their lives. USH2 is the most common form of USH and USH2 patients account for more than 50% of all USH patients [2, 4]. USH3 patients are not born deaf and blind. They usually show a gradual loss of their hearing and vision.

Up to now, 16 genes have been identified that may cause USH (https://sph.uth.edu/retnet/sum-dis.htm), three genes of them (*USH2A* (usherin) [5], *ADGRV1* (Adhesion G Protein-Coupled Receptor V1) [6] and *DFNB31* (autosomal recessive deafness 31) [7]) are the USH2 genes. *USH2A* gene is the major pathogenic gene for USH2 and responsible for more than 74% USH2 cases [8]. *USH2A* gene is located on chromosome 1q41 and has two alternatively spliced isoforms. The shorter *USH2A* isoform was first identified in 1998 [5] and the much longer *USH2A* isoform b was identified by van Wijk et al. in 2004 [9]. To date, all 72 exons of *USH2A* isoform b have been carried out plenty of mutational analyses and found many pathogenic mutations (including splicing mutations at splice sites) [10, 11]. The protein usherin, encoded by the isoform b of *USH2A,* is presumed with 5202 amino acids and anchored on the cell membrane [12]. In mammalian photoreceptors, the usherin are expressed specifically in the connecting cilia and involved in the cargo delivery from the inner segment to the outer segment [13]. Previous research has been shown that mutations of *USH2A* could cause nonsyndromic recessive RP [14, 15]. What is more, *USH2A* gene also related to tactile sensitivity and acuity [16].

In this study, five deleterious variants and 14 non-pathogenic variants in the *USH2A* gene were identified in four Chinese USH2 patients by mutation screening. Two of the pathogenic variants we detected were novel.

## Methods

### Sample collection and ethics statement

Unrelated Chinese patients diagnosed with USH2 were included in this study. Two hundred unrelated normal individuals were recruited in this study as healthy controls. All patients underwent careful clinical examinations in Shanghai Tenth People’s Hospital and Clinical diagnosis of Usher syndrome were based on examination of optical coherence tomography (OCT) and electroretinogram (ERG), the typical RP fundus appearance, intact vestibular function, and sensorineural hearing impairment. The reference sequence from NCBI served as controls. This study was granted approval by the Declaration of Helsinki and approved by the institutional review board (IRB) of Tongji Eye Institute of Tongji University School of Medicine (Shanghai, China). Written informed consent was obtained from all participants.

### The grading system for severity of hearing impairment and evaluation of vestibular function

The severity of hearing impairment can be judged according to the pure tone hearing threshold: mild hearing loss: 26–40 dB HL, moderate hearing loss: 41–80 dB HL, severe hearing loss: > 80 dB HL. Vestibular function tests include position tests and hot and cold water tests. (1) Position test: Dix-Hallpike technique was used to induce dizziness. Keeping the patient horizontal with his head pressed down by 30°. The head and eyes of the patient first turn to the right and then to the left, and repeated it several times to observe the severity and duration of nystagmus and dizziness. (2) Hot and cold water test: otoscopy should be performed before the test, and it can be performed without tympanic membrane perforation. The patient lies on his back and raises his head 30°to keep the lateral semicircular canal becomes upright. Each external ear canal was filled with cold or warm water for 40 s. Discomfort from warm water is usually lighter than cold water. In normal patients, cold water stimulates the nystagmus of the slow-phase stimulus side and the fast phase deviates from the stimulus side; warm water stimulus has the opposite response; in patients with vestibular cochlear nerve and vestibular nucleus disease, irrigation on the lesion side cannot induce nystagmus or nystagmus appears healthy slightly slower or shorter duration.

### Sample preparation and variant screening

Peripheral blood samples from all the participants were collected in EDTA tubes. Standard protocols of RelaxGene Blood DNA System (TianGen, Beijing, China) were used to extract Genomic DNA according to the manufacturer’s instructions. DNA samples were stored at − 80 °C environment before used. Using the Primer3 software (http://primer3.sourceforge.net/) designed specific primers encompassing *USH2A* exons 2 to 72 (Table S1) (including the intron-exon boundary). The coding region was amplified by polymerase chain reaction (PCR) and using Sanger sequencing which was performed using ABI3730 Automated Sequencer (PE Biosystems, Foster City, CA, USA) study alleles. Nucleotide sequences assayed by Sanger sequencing were compared with the published DNA sequence of the *USH2A* gene (NCBI Reference Sequence: NM_206933.3(http://genome.ucsc.edu/cgi-bin/hgc?hgsid=785073911_5XSAy4TZHazZdzeKszSK5wYZ4AfE&g=htcCdnaAli&i=NM_206933&c=chr1&l=215796232&r=216596790&o=215796232&aliTable=refSeqAli)). The cDNA numbering + 1 position corresponds to A in the ATG translation initiation codon for *USH2A*.

### Predictions of the pathogenic effect of missense variations and splice-site

We used several different computer algorithms: SIFT and PROVEAN (http://provean.jcvi.org/genome_submit_2.php), PolyPhen-2 (http://genetics.bwh.harvard.edu/pph2/) and MutationTaster (http://www.mutationtaster.org/) to predict the pathogenic effect of missense variants. Human Splicing Finder (HSF) (http://www.umd.be/HSF3/) was used to predict the pathogenicity of Splicing-site. Evolutionary conservation across species was evaluated through the alignment of orthologous USH2A protein sequences (including Mouse, Troglodyte, Bovine, Chicken, Mulatta and Zebrafish) with the human USH2A protein sequence, using Clustal Omega (https://www.ebi.ac.uk/Tools/msa/clustalo/).

## Results

### Clinical characteristics of the USH2 patients

**Fig. 1:**
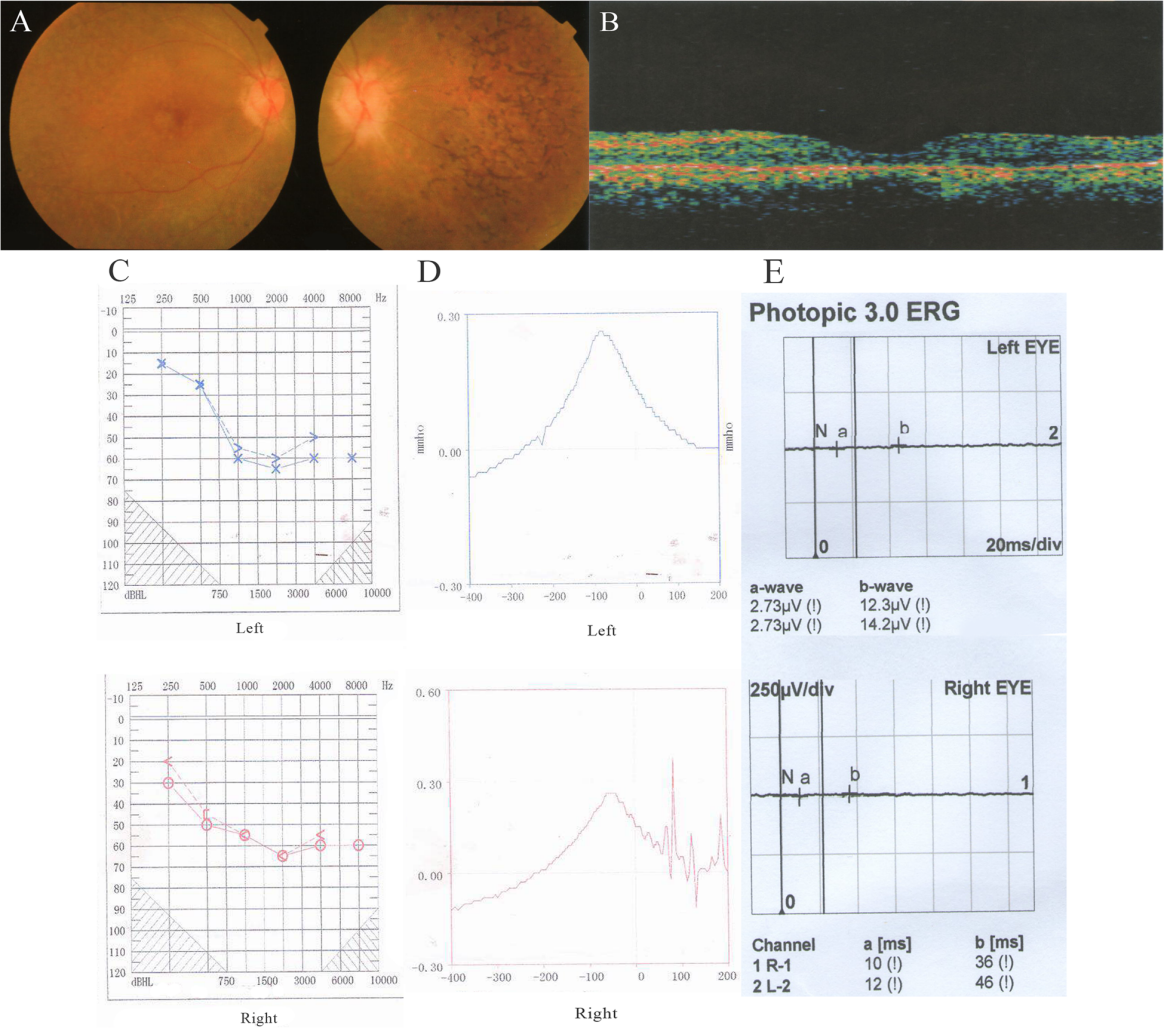
Representative clinical examination of the USH2 patients. **a** The appearance of the fundus of patient No.003 shows typical retinal degeneration including irregular pigment clumps in the retina and attenuation of the retinal vessels. **b** OCT of left eye of patient No.002. **c** Result of pure tone audiogram testing of patient No.002 indicated bilateral hearing loss, cross or circle labels indicate air-conduction hearing, and right angle labels indicate bone-conduction hearing. **d** Tympanogram of patient No.003 demonstrated limited sound system activity of the middle ear. **e** The results of ERG of patient No.003 displayed indistinguishable wave amplitude

According to the data of their families, all the recruited patients followed the pattern of autosomal recessive inheritance. Representative fundus photographs indicated typical RP features (Fig. 1a), and representative OCT imaging suggested significantly diminished retinal thickness in patients (Fig. 1b). Moreover, most patients have moderate-to-severe hearing impairment, and analysis of pure tone audiogram testing demonstrated bilateral decrease of air-conduction and bone-conduction auditory (Fig. 1c). The tympanograms were showed type As which means limited activity of the middle ear transmission system (Fig. 1d). The ERG wave amplitude of patients were undetectable (Fig. 1e). Those features indicate the diagnosis of USH2, and detailed clinical information of the patients is summarized in Table 1.

**Table 1:** The clinical information of the patients

Patient number	Gender	Diagnosis	Inheritance pattern	Onset age	ERG	Fundus appearance	Hearing impairment	Vestibular function	Night blindness
001	M	USH2	autosomal recessive	21	no reaction	RP	Severe	Normal	Yes
002	M	USH2	autosomal recessive	21	no reaction	RP	Moderate	Normal	Yes
003	F	USH2	autosomal recessive	19	no reaction	RP	Moderate	Normal	Yes
004	F	USH2	autosomal recessive	23	no reaction	RP	Severe	Normal	Yes

### Pathogenicity analysis of the USH2A variants

In this study, we found 19 changes among four USH2 patients by exon sequencing of the USH2A gene. According to the result of computer algorithms, five of them were predicted to be pathogenic variants (Table 2). All the other 14 variants predicted non-pathogenic are listed in the Table S2.

**Table 2:** Identified pathogenic variants in *USH2A* gene in this study and their prediction results from the analysis programs

Patient number	Exon/Intron	Nucleotide change	Amino acid change	Type	MutationTaster	SIFT	PROVEAN	PolyPhen-2	HSF	Ref.
001	IVS42	c.8559-2A > G	–	Heterozygous	Disease causing	–	–	–	Potential alteration of splicing	[17]
002	EX19	c.4217C > A	p.Ser1406X	Heterozygous	Disease causing	Damaging	Deleterious	–	–	Novel
002	EX61	c.11780A > G	p.Asp3927Gly	Heterozygous	Disease causing	Tolerated	Deleterious	Probably damaging (Score 0.911)	–	Novel
003	IVS58	c.11389 + 3A > T	–	Heterozygous	Disease causing	–	–	–	Potential alteration of splicing	SCV000579424.1
004	EX42	c.8232G > C	p.Trp2744Cys	Heterozygous	Disease causing	Damaging	Deleterious	Probably damaging(Score 1.000)	–	[18]
004	IVS42	c.8559-2A > G	–	Heterozygous	Disease causing	–	–	–	Potential alteration of splicing	[17]

These five heterozygous mutations include a nonsense mutation (c.4217C > A (p.Ser1406X)), two splice site mutations (c.8559-2A > G and c.11389 + 3A > T), and two missense mutations (c.8232G > C (p.Trp2744Cys) and c.11780A > G (p.Asp3927Gly)). All of these can be predicted as harmful by the computer prediction tool.

In the five pathogenic variants, two of them (c.4217C > A (p.Ser1406X) and c.11780A > G (p.Asp3927Gly)) were novel (can not be found in the variants in publicly available human genome aggregation data sets) and three (c.8559-2A > G, c.8232G > C (p.Trp2744Cys) and c.11389 + 3A > T) were reported. All the variants predicted to be pathogenic were absent in 200 Chinese unrelated healthy controls.

**Fig. 2:**
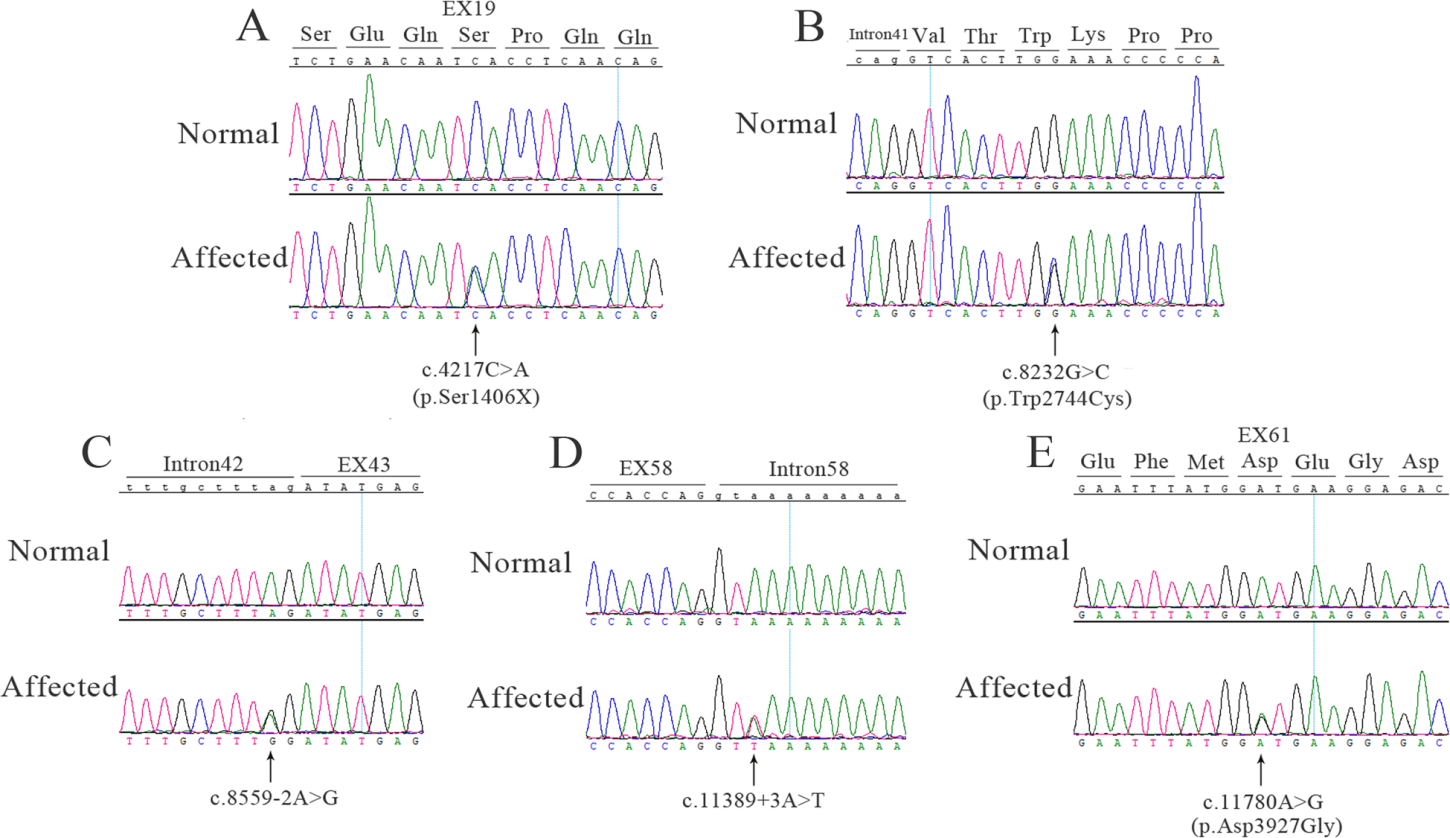
Direct sequencing analysis of the pathogenic variants in *USH2A* identified in this study. **a** Sequence shown the heterozygous nonsense variant c.4217C > A (p.Ser1406X) and the corresponding wild-type sequence. **b** Sequence shown the heterozygous missense variant c.8232G > C (p.Trp2744Cys) and the corresponding wild-type sequence. **c** Sequence shown the heterozygous one-base-substitution variant c.8559-2A > G and the corresponding wild-type sequence. **d** Sequence shown the heterozygous one-base-substitution variant c.11389 + 3A > T and the corresponding wild-type sequence. **e** Sequence shown the heterozygous missense variant c.11780A > G (p.Asp3927Gly) and the corresponding wild-type sequence. Arrows indicate the position of variants

**Fig. 3:**
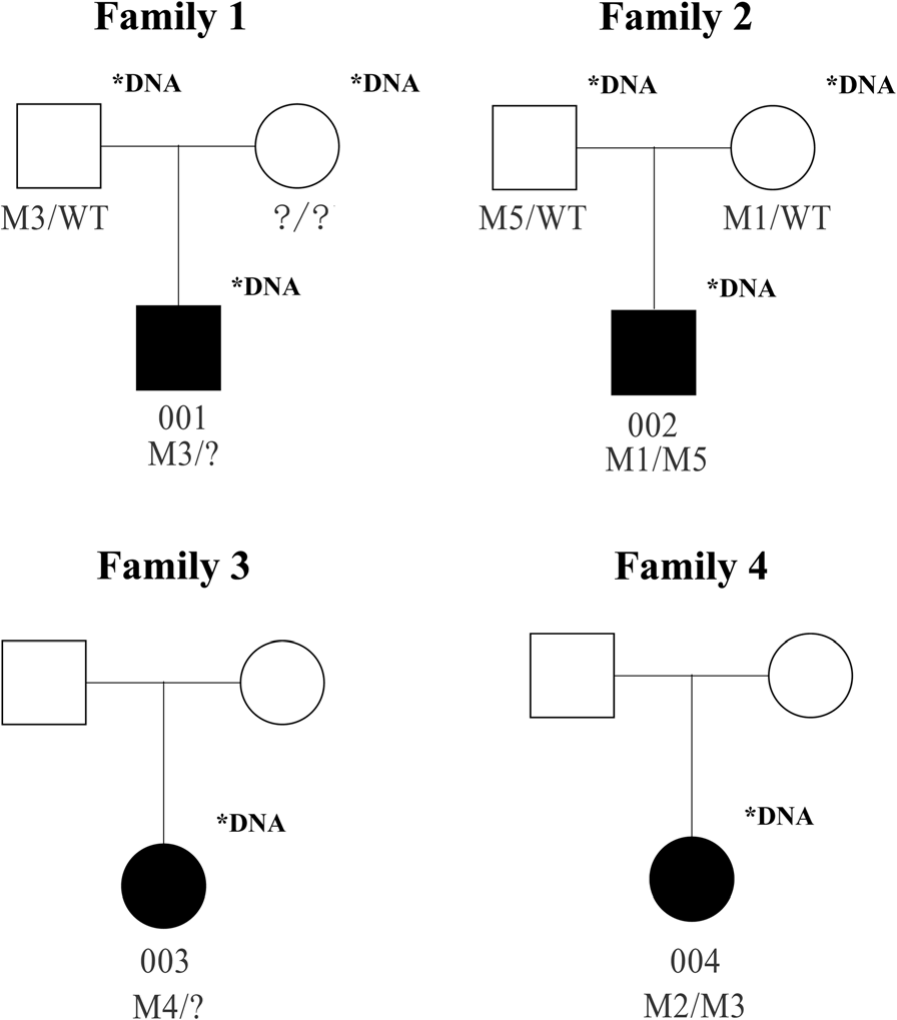
Pedigree of the Chinese Usher syndrome type II patients’ family. The black filled shapes mean individuals diagnosed with USH2 and the unfilled mean unaffected ones. Males are represented by squares, females circles. Patient number is below the individuals’ symbol. Individuals with available DNA samples were marked with asterisk. Question mark means the unknown allelic variant. M1: c.4217C > A (p.Ser1406X); M2: c.8232G > C (p.Trp2744Cys); M3: c.8559-2A > G; M4: c.11389 + 3A > T; M5: c.11780A > G (p.Asp3927Gly)

Two novel variants (c.4217C > A (p.Ser1406X) (Fig. 2a) in exon 19 and c.11780A > G (p.Asp3927Gly) (Fig. 2e) in exon 61) were found in patient No.002. In the family of patient No.002, c.4217C > A (p.Ser1406X) was found in his mother (Figure S1 B) and c.11780A > G (p.Asp3927Gly) was found in his father (Figure S1 C). Parents of patient No.002 are normal. Reported intron sequence variant c.8559-2A > G (Fig. 2c) and missense variant c.8232G > C (p.Trp2744Cys) (Fig. 2b) in exon 42 were found in patient No.004. Interestingly, intron sequence variant c.8559-2A > G also found in patient No.001 and his unaffected father (Figure S1 A). Finally, an intron sequence variant c.11389 + 3A > T (rs753886165) (Fig. 2d) was found in patient No.003. However, in patient No.001 and No.003, we do not find the allelic variant in the *USH2A* gene. The pedigrees of the four patients with variants in *USH2A* are shown in the Fig. 3.

**Fig. 4:**
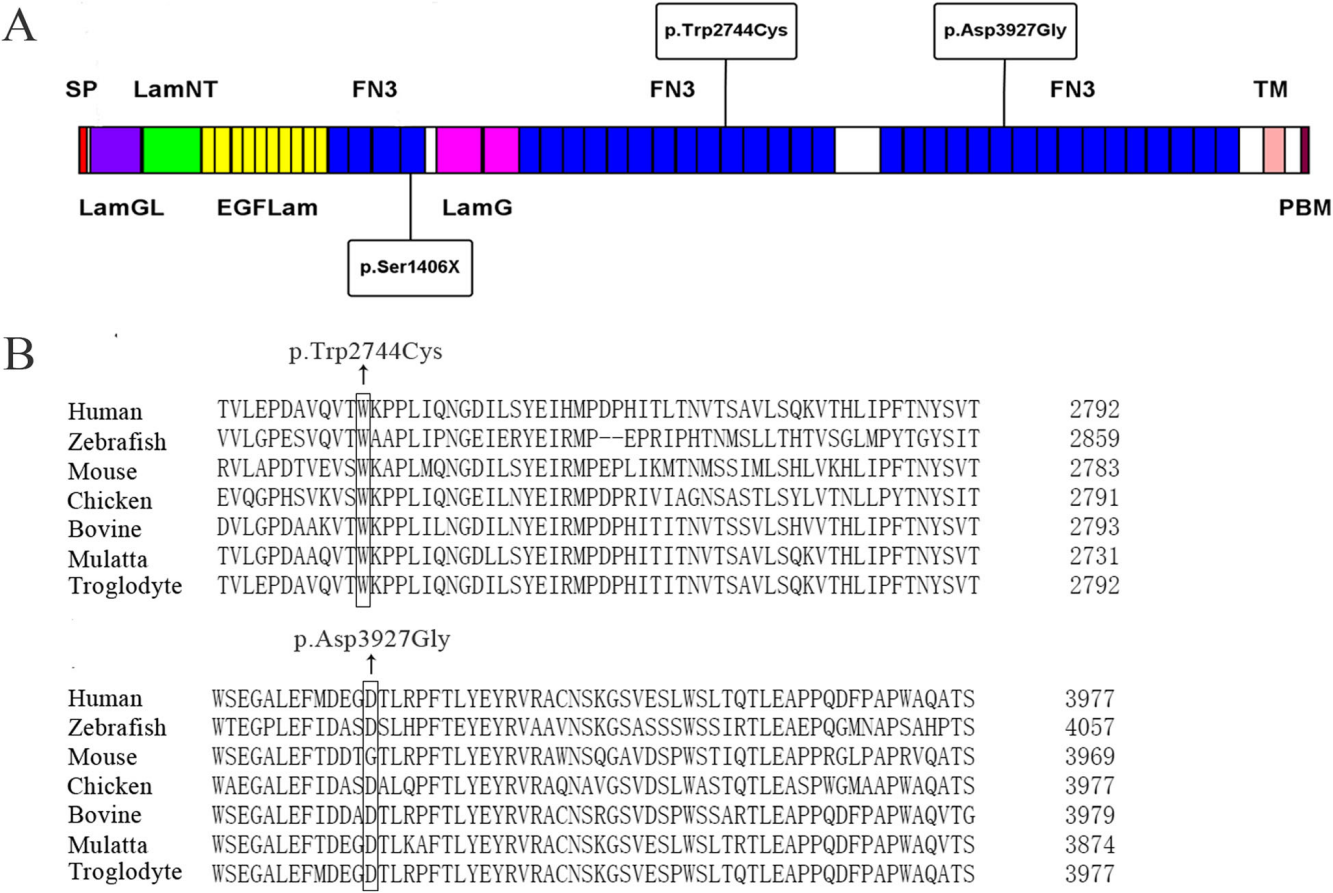
**a** Schematic illustration of the exon pathogenic variants identified in this study along the *USH2A* isoform b protein domains. SP: signal peptide; Lam GL: Laminin G-like domain; Lam NT: Laminin N-terminal; EGF Lam: EGF-like domain; FN3: fibronectin type-III; LamG: Laminin G domain; TM: transmembrane region; PDB: PDZ-binding domain **b** Amino acid sequence alignments obtained by Clustal Omega software. Exon missense mutations in this study p.Trp2744Cys (c.8232G > C) and p.Asp3927Gly (c.11780A > G) in Human *USH2A* gene aligned with other species including Troglodyte, Zebrafish, Chicken, Mulatta, Mouse and Bovine

For the exon pathogenic variants identified in this study, we examined the location of them along the usherin. Finally, we identified functional domains the exon variants located within (Fig. 4a). Additionally, we aligned *USH2A* sequences among different species, including Human, Troglodyte, Mulatta, Bovine, Chicken, Mouse and Zebrafish for each of the two novel missense variants by Clustal Omega. The results of the conservative analysis of amino acid sequences were shown in Fig. 4.

## Discussion

Currently, 16 genes associated with USH have been identified, and three are USH2-causing gene. The *USH2A* gene causes 30–40% of USH2 cases and 10–15% of recessive RP cases [19]. Usherin is localized to a spatially restricted membrane microdomain in mammalian photoreceptors [13]. Previous researches have shown that congenital usherin protein mutations can induce the connecting cilium disorder and eventually lead to blindness [20].

Up to now, mutation screening in Chinese patients was revealed 25 mutations in previous researches [15, 18, 21–24]. In Southern population of China, 8.47% of sporadic RP patients are belong to USH [21]. In this study, we identified two novel variants (a missense variant and a nonsense variant) in the *USH2A* gene of four Chinese patients diagnosed with USH2 and found three reported mutations.

Isoform b of *USH2A* consists 8 domains, including N-terminal signal peptide (SP), laminin G-like domain (Lam GL), laminin N-terminal (Lam NT), laminin-type EGF-like domain (EGF Lam), fibronectin Type III (FN3), laminin G domain (LamG), transmembrane region (TM), and a PDZ-binding motif (PBM) at its C-terminal end [9]. By the PBM interacted with the PDZ domain of harmonin and whirlin, USH2A integrated into the USH protein network [25].

All of the two novel pathogenic variants are located in the FN3 domain (Fig. 4a). c.4217C > A (p.Ser1406X) is located in the fourth FN3 domain, and c.11780A > G (p.Asp3927Gly) is located in the 24th FN3 domain. In addition, reported mutation c.8232G > C (p.Trp2744Cys) is located in the fourteenth FN3 domain.

Heterozygous nonsense variant c.4217C > A (p.Ser1406X) causing a premature stop codon at 1406 is located on exon 19, and leads to a subsequent loss of 3796 amino acids, which make the protein usherin to lose more than 70% of its amino acids including 30 TM domains, 2 LamG domains, TM domain, and PBM domain. Therefore, heterozygous nonsense variant c.4217C > A (p.Ser1406X) affecting the structure and function of the protein usherin have a great possibility of causing the USH2. Novel missense variant p.Asp3927Gly (c.11780A > G) replaces a polaraspartic acid with a nonpolar hydrophobic glycine at codon 3927. Amino acid substitutions caused by reported missense variant p.Trp2744Cys (c.8232G > C) occur at highly conserved sites among the tested species. Interestingly, sites of novel missense variant p.Asp3927Gly (c.11780A > G) in the Human, Troglodyte, Mulatta, Chicken, Zebrafish and Bovine are conserved while the Mouse not.

For the Family # 2 and Family # 3, the following possibilities could be attributed to the unknown allelic variants: 1. Variants in deep-intronic regions of *USH2A* were not detected because this part of the genome was not covered in the screening. 2. Variants in regulatory elements except the *USH2A* gene cannot be excluded. 3. The duplication or deletion of other alleles may not be detected due to the absence of copy number variation analysis.

Because of unknown allelic variants in Family # 2 and Family # 3, we suppose that other pathogenic variants may exist in patients. Data from Family # 2 was supportive for the pathogenicity of the novel nonsense variant c.4217C > A (p.Ser1406X) and novel missense variant c.11780A > G (p.Asp3927Gly). The other three pathogenic variants are known pathogenic mutations that have been reported. However, sufficient biological and clinical evidence was required to reveal the relationship between the identified variants and the USH2. The detailed reasons of these pathogenic mutations leading to visual defects and hearing impairment have not been elucidated, and pending further function and mechanism investigations.

In all the three USH2-causing genes, *USH2A* gene is the most important causative gene, and the usherin which encoded by *USH2A* is crucial for the long-term maintenance of mammalian photoreceptors [13]. Accordingly, identification of the mutations in the *USH2A* gene will not only elucidate the role of *USH2A* in USH2, but also aid the clinical diagnosis and help to find effective treatments for USH2.

## Conclusions

In conclusion, we have described five heterozygous variants may cause USH2 in *USH2A* in four Chinese patients with USH2, two of which were novel. The specific mechanism for these variants to induce USH2 needs further research to confirm. The findings in this study expand the spectrum of *USH2A* mutations in USH.

## Supplementary information

**Additional file 1: Table S1.** Primer information for the USH2A gene exons 2 to 72 sequencing. (XLS 31 kb)

**Additional file 2: Table S2.** variants predicted non-pathogenic in this study.

**Additional file 3: Figure S1.** Sequencing data of variants c.8559-2A>G, c.4217C>A (p.Ser1406X) and c.11780A>G (p.Asp3927Gly) identified in the father of patient No.001 and the parents of patient No.002.

## Data Availability

All data generated or analyzed during this study are included in this published article.

## Abbreviations

ADGRV1: Adhesion G protein-coupled receptor V1
DFNB31: Autosomal recessive deafness 31
EGF Lam: Laminin-type EGF-like domain
ERG: Electroretinogram
FN3: Fibronectin Type III
HSF: Human splicing finder
IRB: Institutional review board
Lam GL: Laminin G-like domain
Lam NT: Laminin N-terminal
LamG: Laminin G domain
OCT: Optical coherence tomography
PBM: PDZ-binding motif
PCR: Polymerase chain reaction
RP: Retinitis pigmentosa
SP: Signal peptide
TM: Transmembrane region
USH: Usher syndrome
USH1: Usher syndrome type I
USH2: Usher syndrome type II
USH2A: Usherin
USH3: Usher syndrome type III
