# Supplementary figures and images for: Mutation screening of the USH2A gene reveals two novel pathogenic variants in Chinese patients causing simplex usher syndrome 2
Source: BMC Ophthalmol. 2020 Feb 24;20:70. doi: 10.1186/s12886-020-01342-y (PMC7038606; doi:10.1186/s12886-020-01342-y)

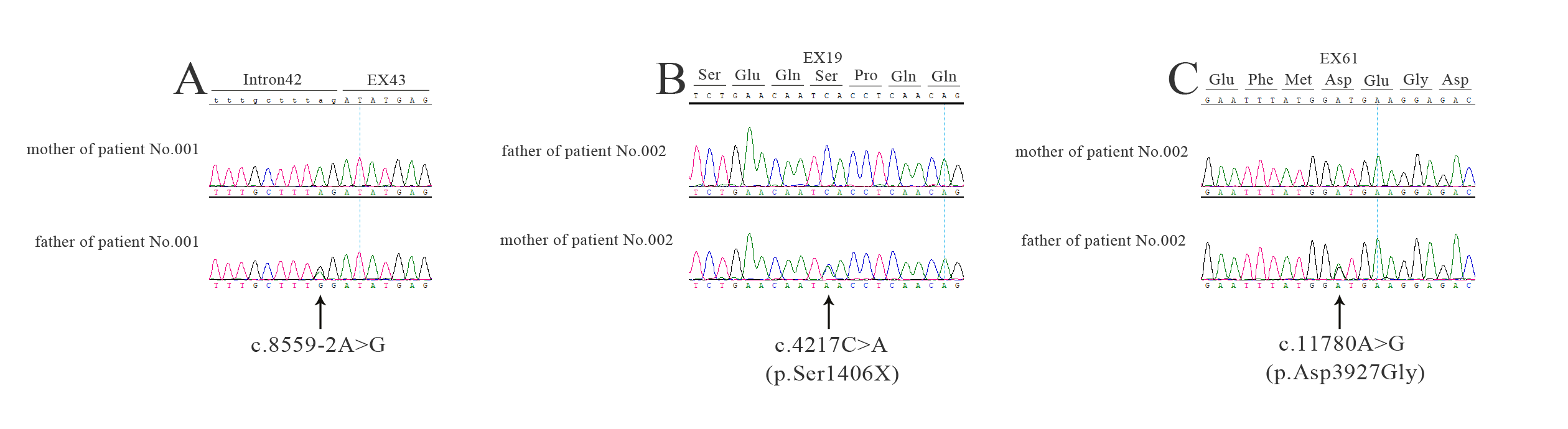

Supplement: Supplementary file 3 — Additional file 3: Figure S1. Sequencing data of variants c.8559-2A>G, c.4217C>A (p.Ser1406X) and c.11780A>G (p.Asp3927Gly) identified in the father of patient No.001 and the parents of patient No.002. [file 12886_2020_1342_MOESM3_ESM.tif]
